# Evaluating a Multidisciplinary Team Approach to Elder Abuse

**DOI:** 10.1093/geroni/igab046.1057

**Published:** 2021-12-17

**Authors:** Carmen Morano, Erin Berical

**Affiliations:** 1 SUNY-Albany, Albany, New York, United States; 2 University at Albany, Albany, New York, United States

## Abstract

This paper presents findings from a University and Community-based Agency collaboration to design and implement a preliminary evaluation of the Elder Abuse Multidisciplinary Team (E-MDT) Intervention. This intervention brings professionals from a variety of fields to investigate and respond to elder abuse. Data from 22 Interviews with staff along with anonymous survey data from E-MDT team members/staff (n=312) sought to establish team successes, challenges in implementation, and ongoing functioning. Themes that emerged in creating successful teams include: Establishing Buy-In and Trust of the team members, The Benefit of sharing experience and practical knowledge with other program sites; and Recognizing the Differences related to Onboarding and Sustaining New programs versus Sustaining Existing Programs. Themes related to responding during COVID revealed challenges such as Adapting to Technology and Inconsistent Access to the Internet. It was noted that remote meetings were easier to attend than face-to-face meetings. Data from the survey found the vast majority of respondents view the E-MDTs as having a positive impact on Clients (93%); while 93% of respondents indicated a positive impact on their Approach to Practice and the service area of their agency. Approximately 80% of the respondents indicated their multidisciplinary teams were Effective. Responses to 3-Open Ended questions included in the survey echoed similar themes from the interviews, as well as comments about their Professional Development and the complexity of responding to elder abuse. The paper will close with a discussion of the strategies used to facilitate the collaboration and complete the evaluation during the COVID-19 pandemic.

